# Sequencing and Analysis of Full-Length cDNAs, 5′-ESTs and 3′-ESTs from a Cartilaginous Fish, the Elephant Shark (*Callorhinchus milii*)

**DOI:** 10.1371/journal.pone.0047174

**Published:** 2012-10-08

**Authors:** Yue Ying Tan, Rimantas Kodzius, Boon-Hui Tay, Alice Tay, Sydney Brenner, Byrappa Venkatesh

**Affiliations:** 1 Comparative Genomics Laboratory, Institute of Molecular and Cell Biology, Agency for Science, Technology and Research, Singapore, Singapore; 2 Department of Paediatrics, Yong Loo Lin School of Medicine, National University of Singapore, Singapore, Singapore; Auburn University, United States of America

## Abstract

Cartilaginous fishes are the most ancient group of living jawed vertebrates (gnathostomes) and are, therefore, an important reference group for understanding the evolution of vertebrates. The elephant shark (*Callorhinchus milii*), a holocephalan cartilaginous fish, has been identified as a model cartilaginous fish genome because of its compact genome (∼910 Mb) and a genome project has been initiated to obtain its whole genome sequence. In this study, we have generated and sequenced full-length enriched cDNA libraries of the elephant shark using the ‘oligo-capping’ method and Sanger sequencing. A total of 6,778 full-length protein-coding cDNA and 10,701 full-length noncoding cDNA were sequenced from six tissues (gills, intestine, kidney, liver, spleen, and testis) of the elephant shark. Analysis of their polyadenylation signals showed that polyadenylation usage in elephant shark is similar to that in mammals. Furthermore, both coding and noncoding transcripts of the elephant shark use the same proportion of canonical polyadenylation sites. Besides BLASTX searches, protein-coding transcripts were annotated by Gene Ontology, InterPro domain, and KEGG pathway analyses. By comparing elephant shark genes to bony vertebrate genes, we identified several ancient genes present in elephant shark but differentially lost in tetrapods or teleosts. Only ∼6% of elephant shark noncoding cDNA showed similarity to known noncoding RNAs (ncRNAs). The rest are either highly divergent ncRNAs or novel ncRNAs. In addition to full-length transcripts, 30,375 5′-ESTs and 41,317 3′-ESTs were sequenced and annotated. The clones and transcripts generated in this study are valuable resources for annotating transcription start sites, exon-intron boundaries, and UTRs of genes in the elephant shark genome, and for the functional characterization of protein sequences. These resources will also be useful for annotating genes in other cartilaginous fishes whose genomes have been targeted for whole genome sequencing.

## Introduction

Cartilaginous fishes are the most basal phylogenetic group of living jawed vertebrates (gnathostomes). They shared a common ancestor with bony vertebrates (comprising ray-finned fishes, lobe-finned fishes, and tetrapods) approximately 450 million years ago (Mya) [1]. To date, approximately 1,000 extant species of cartilaginous fishes have been described [2], and divided broadly into two groups: the holocephalans (chimaeras) and elasmobranchs (sharks, rays and skates). The two groups diverged ∼420 Mya [3] and thus represent distinct lineages that have been evolving independently over an evolutionary period longer than that between mammals and amphibians (330 million years). Because of their unique phylogenetic position, cartilaginous fishes constitute a critical group for our understanding of the origin and evolution of vertebrates. Consequently, several cartilaginous fishes have been targeted for whole-genome sequencing and comparative genomic studies (see Bernardi et al., 2012[4]). For example, the elephant shark (*Callorhinchus milii*), a holocephalan chimaera, was identified as a model cartilaginous fish genome because of its relatively small genome size (∼910 Mb) [5] and sequenced to 1.4× coverage by Sanger sequencing [6]. Comparison of this low-coverage sequence with bony vertebrate genomes indicated that elephant shark and human share a higher proportion of conserved synteny and conserved sequences than teleost fishes and human [6], [7]. Currently efforts are underway to obtain a whole-genome assembly of the elephant shark (http://esharkgenome.imcb.a-star.edu.sg/). Among elasmobranchs, the little skate (*Leucoraja erinacea*) has been sequenced to 26× coverage using the short-read Illumina GAIIx platform [8].

In addition to genome sequence, ESTs have also been sequenced from several cartilaginous fishes. Parton et al. sequenced 31,167 and 32,562 ESTs from the embryonic cell lines of the little skate and the spiny dogfish shark (*Squalus acanthias*), respectively [9]. In order to identify Hox genes in the small-spotted catshark (*Scyliorhinus canicula*), 225,580 ESTs were sequenced from embryos and tissues at various stages of development [10]. In addition, 107,231, 103, 996 and 92,334 ESTs were sequenced from the embryos of the small-spotted catshark (stage 24–30), the little skate (stage 20–29) and the elephant shark (stage 32), respectively [8]. Embryonic ESTs have also been sequenced (165,819 ESTs) for the cloudy catshark, *Scyliorhinus torazame* [11]. More recently, ∼10,000 ESTs were sequenced from the electric organ, a specialized organ of the Pacific electric ray, *Torpedo californica* [12]. Sequencing ESTs is an efficient strategy for discovering genes and for profiling expression pattern of genes, even in the absence of genome sequence for a species. ESTs also allow precise demarcation of exon-intron boundaries and identification of splice variants. However, ESTs are typically short (200 to 600 bp) and do not include the entire coding sequence and the UTRs. Thus they are of limited use in predicting protein sequences and UTRs. An alternative strategy that can overcome these limitations is the cloning and sequencing of full-length cDNA sequences. In addition to facilitating the annotation of protein-coding sequences, exon-intron boundaries, alternative exons, transcription start sites and UTRs, full-length cDNA clones are also valuable for expressing proteins and generating mutant clones that can shed light on the function of proteins. Generation of full-length cDNA sequences from human, mouse, chicken and *Xenopus* (*X. tropicalis* and *X. laevis*) (e.g., [13], [14], [15], [16], [17]) has facilitated the prediction of a comprehensive set of expressed genes in their genomes besides refining the annotation of exon-intron boundaries and the discovery of alternative transcripts. Among bony fishes (ray-finned fishes), about 9,000 full-length cDNA clones have been sequenced from the Atlantic salmon (*Salmo salar*) [18], [19]. A large number of full-length cDNA sequences have also been generated from the channel catfish (*Ictalurus punctatus*) and the blue catfish (*Ictalurus furcatus*) [20]. These full-cDNA sequences would be invaluable for annotating the whole genome sequences of the Atlantic salmon and channel catfish that are currently being sequenced (see [4]).

In this study we have used the ‘oligo-capping’ method [21] to clone and sequence full-length cDNA sequences from six tissues of the adult elephant shark by Sanger sequencing method. Full-length cDNA libraries were prepared from gills, intestine, kidney, liver, spleen and testis. In cartilaginous fishes, spleen is the major site of immune cell production. Indeed, cartilaginous fishes are the oldest living group of vertebrates that possess this specialized organ associated with adaptive immunity. In total, 17,479 full-length coding and noncoding cDNAs were sequenced from the six tissues of the elephant shark. In addition, a total of 30,375 5′ESTs and 41,317 3′ESTs were also sequenced. Although 5′ and 3′ESTs do not code for full-length protein sequences, they are extremely useful in mapping transcription start sites, 5′UTRs and 3′UTRs in the genome context. The functional annotation of full-length coding cDNA, 5′EST and 3′EST sequences were carried out by analysing Gene Ontology (GO) terms and protein domains associated with them. The cDNA clones and sequences generated in this study are useful resources for annotating genes in elephant shark and other cartilaginous fish genomes and for functional studies of elephant shark genes.

## Methods

### Ethics statement

The extraction of RNA from frozen fish samples in our lab is approved by the Institutional Animal Care and Use Committee (IACUC) of the Institute of Molecular and Cell Biology.

### Tissue collection and RNA extraction

Adult elephant sharks were collected at Western Port Bay, Victoria in Australia. Tissues were flash-frozen in liquid nitrogen, transported to Singapore in dry ice and stored at −80°C until RNA extraction. Total RNA was isolated from various tissues using TRIzol reagent (Invitrogen, USA) according to the manufacturer’s protocol. 150 µg of total RNA from each tissue was treated with 100 U of DNase I (Roche) and 80 U RNaseOUT Recombinant Ribonuclease Inhibitor (Invitrogen) for 30 min at 37°C and purified by RNeasy Mini Kit (Qiagen).

### cDNA library construction

DNAse treated total RNA was used for making libraries enriched for full-length cDNA by the ‘oligo-capping’ method developed by Suzuki and Sugano [21]. Dephosphorylation of the 5′-end of the non-capped truncated RNA was performed using Bacterial Acid Phosphatase (Takara) in the presence of RNaseOUT, thereby rendering them incapable of ligating to the RNA oligonucleotide. Decapping of full-length RNA was performed with Tobacco Acid Pyrophosphatase (Epicentre) leaving a phosphate at the 5′-ends of full-length mRNA. Finally, the 5′-amino modified RNA oligonucleotide (AGCAUCGAGUCGGCCUUGUUGGCCUACUGG) was ligated to the 5′-ends of decapped mRNA using T4 RNA ligase (TaKaRa) and RNaseOUT. The oligo-capped RNA was treated with DNaseI to ensure digestion of any residual genomic DNA in the preparation. Each of these reactions was followed by a step of phenol/chloroform extraction and ethanol precipitation. The oligo-capped RNA was enriched for polyadenylated RNA using Oligo dT Cellulose (Ambion). The first strand cDNA was synthesized using poly(T) oligo (GCGGCTGAAGACGGCCTATGTGGCCT_17_V) and Superscript II Reverse Transcriptase (Invitrogen) in an overnight reaction at 42°C. The RNA template was then degraded in 15 mM NaOH at 65°C for 40 min and the single-strand cDNA (ssDNA) was purified by an Illustra MicroSpin™ S-400 HR column (GE Healthcare). The ssDNA was used as a template to synthesize double-stranded DNA (dsDNA) by PCR with TaKaRa LA Taq™ (TaKaRa) and the primer pair, RK024 (GCGGCTGAAGACGGCCTATGT) and RK074 (GCATCGAGTCGGCCTTGTTGGCCTACTG). The PCR cycle profile comprised 12 cycles of 94°C for 1 min, 58°C for 1 min and 72°C for 10 min. The resultant dsDNA was purified by phenol/chloroform extraction and ethanol precipitation.

The dsDNA was digested with *Sfi*I (Fermentas) at 50°C overnight followed by phenol/chloroform extraction and ethanol precipitation. The *Sfi*I-digested dsDNA was size fractionated by agarose gel electrophoresis. Fragment sizes of ∼0.5–1.6 kb and ∼1.6–3.5 kb were extracted and purified by QIAquick Gel Extraction Kit (Qiagen) and ethanol precipitation. The fractions were ligated separately to a pBS/SfiI vector (modified pBS by additing *SfiI* sites) using DNA Ligation Kit Ver.1 (TaKaRa) at 16°C overnight. The ligation mixtures were transformed into ElectroMAX™ DH10B™ cells (Invitrogen) by electroporation. Recombinant clones were picked into 384-well plates and their plasmid DNAs were extracted using 96-well Plasmid Kit (Geneaid).

### Sequencing and sequence analysis

Sequencing was performed with BigDye® Terminator v3.1 Cycle Sequencing Kit (Applied Biosystems) on ABI 3730 Automated DNA sequencers using the vector primers, M13FL (CGTTGTAAAACGACGGCCAGTG) and KSML (GGGAACAAAAGCTGGGTACC). The ABI trace reads were base-called using PHRED [22], [23] and trimmed for vector sequences and poor quality bases (PHRED score <20). The 5′-end reads were scanned using in-house Perl scripts for the presence of oligo-cap. Polyadenylation tails in the 3′-end reads were detected through pattern search. Sequences with read lengths less than 150 bp were discarded as low quality short-reads. The remaining reads were searched for sequences containing potential repetitive elements by using RepeatMasker (version 3.3.0) and such sequences were discarded. Transcripts of mitochondrial origin were identified by aligning the reads (BLASTN; *E*<10^−30^) with elephant shark mitochondrial genome (accession number HM147137) and discarded. Finally, the sequence reads were checked for any residual vector sequences by searching against UniVec at NCBI using BLASTN and vector sequences, if any, were deleted.

### Selection of clones for generating full-length cDNA

**Figure 1:**
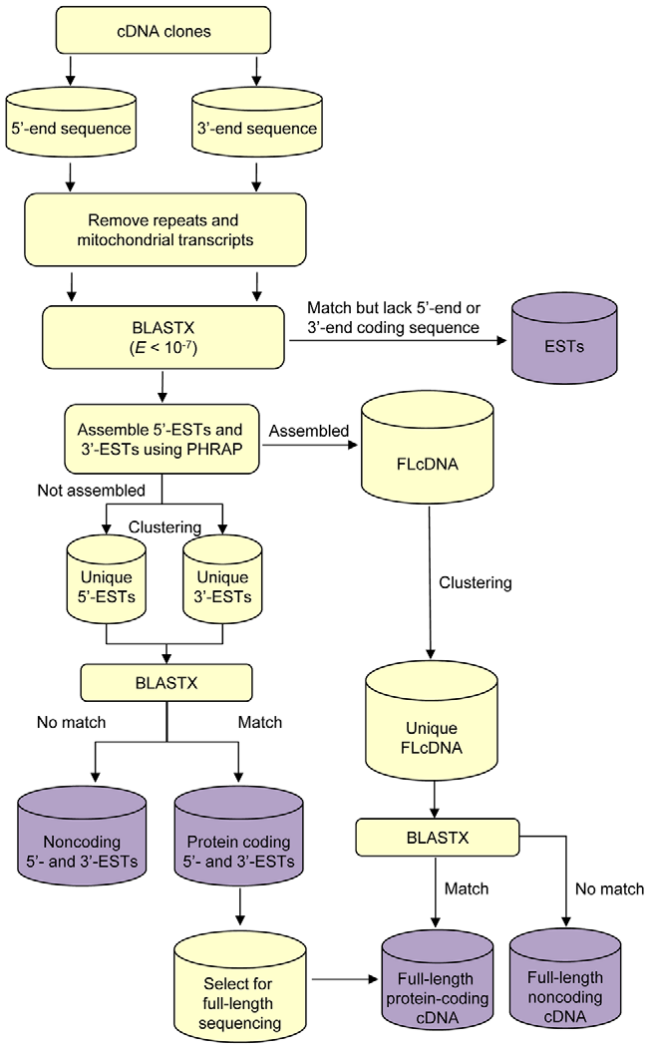
Elephant shark cDNA sequence analysis pipeline.

We first searched all the sequences against the non-redundant (nr) protein database of NCBI using BLASTX (*E*<10^–7^) to identify 5′ or 3′-truncated protein-coding transcripts and classified them as “ESTs” (see Figure 1). The remaining 5′-end sequences including those that did not have protein hits were assembled with their respective 3′-end sequences using PHRAP [24]. Contigs of sequences that assembled with >50% overlap formed the full-length cDNA transcripts. These transcripts were searched against nr protein database at NCBI using BLASTX and those with protein hits were classified as full-length protein-coding cDNAs. The remaining sequences were classified as full-length noncoding cDNAs. These full-length sequences were typically shorter than 800 bp. 5′-end and 3′-end sequences that did not assemble were clustered using ‘cd-hit-est’ tool in the CD-HIT suite [25] with 99% global sequence identity, 95% alignment coverage of the shorter sequence relative to the cluster seed to generate unique sets of 5′-end sequences and 3′-end sequences. These 5′ and 3′-end sequences were again searched against the nr protein database to select pairs of 5′-end and 3′-end sequences that coded for known protein sequences. 5′-end reads of such sequences were sorted according to length and the top ∼800 were selected for generating full-length protein-coding cDNA by primer walking. Walking primers were designed using the program Primer3 [26]. From among the remaining 5′ and 3′-end sequences, transcripts less than 300 bp long were filtered off and the rest retained as 5′-ESTs and 3′-ESTs, respectively.

### Prediction of protein sequences for full-length protein-coding cDNA

The full-length coding sequences were first searched against the NCBI nr protein database using BLASTX (*E*<10^−7^) to identify cDNAs with similarity to known proteins. Any matches to reverse transcriptase were discarded as they could be generated by retroelements. Since the cDNA sequences were generated using the oligo-capping method, the proteins should be encoded in the sense strand of the transcript. Hence any protein matches on the anti-sense strand of the transcript were filtered and classified as non-coding transcripts. The remaining cDNAs were predicted for open reading frames using an in-house Perl script that reads in BLASTX alignments. The resulting protein sequences were searched against the nr protein database using BLASTP (*E*<10^−7^) for confirmation.

### Generation of non-redundant full-length cDNA

The predicted full-length protein sequences were clustered using ‘cd-hit’ [25] with 98% global sequence identity and 90% alignment coverage to obtain a non-redundant set of proteins. To generate a non-redundant set of full-length noncoding sequences, full-length noncoding sequences were clustered using ‘cd-hit-est-2d’ [25] with 98% global sequence identity and 95% alignment coverage. This clustering step was performed together with full-length protein-coding transcripts to identify and exclude any noncoding transcripts that represented UTRs of protein-coding transcripts.

### Polyadenylation signal detection

We searched for the canonical and alternative polyadenylation signals using regular pattern matching scripts. Only signals located between 5 bp and 30 bp upstream of the polyadenylation tail were considered. Polyadenylation signals were identified by the order of precedence as presented by Scheetz *et* *al*. [27].

### Functional annotation

To obtain GO terms for the protein sequences encoded by full-length cDNAs, the protein sequences were searched against the nr protein database at NCBI using BLASTP (*E*<10^−7^). The protein identifiers of their top hits were converted to UniProt identifiers using the ID Mapping web tool (http://www.uniprot.org/). GO terms of these recorded UniProt identifiers were obtained using QuickGO [28]. The number of occurrences of the GO terms was recorded.

To obtain GO terms for the 5′-ESTs and 3′-ESTs, their open reading frames were predicted based on BLASTX (*E*<10^−7^) alignments against nr proteins. These sequences represent partial protein sequence. The partial sequences were searched against the nr protein database using BLASTP (*E*<10^−7^). The protein accession numbers of their top hits were converted to UniProt identifiers. GO terms were then obtained from multispecies UniProt gene association file (GAF version 2.0) [29]. To avoid double counting of GO terms for 5′-EST and 3′-EST pairs from the same clone, GO terms were counted only for their 5′-ESTs. Enrichment of GO terms among 5′-ESTs and 3′-ESTs were analyzed by running customized Perl scripts obtained from the GOstat package [30]. InterPro domains of protein hits for full-length cDNA and ESTs were retrieved by mapping their GO terms to InterPro2GO (version date 31^st^ March 2012) [31].

To obtain KEGG (Kyoto Encyclopedia of Genes and Genomes) orthology (KO) terms for the full-length protein-coding cDNA, they were searched against a manually curated KEGG GENES database using KEGG Automatic Annotation Server (KAAS) [32]. Bi-directional best hit method was applied for assigning KO terms to each query sequence. KEGG orthology assignment was performed based on KEGG GENES from human, mouse, dog, opossum, platypus, chicken, zebra finch, *Xenopus tropicalis* and zebrafish. The KO terms were further searched against the KEGG BRITE database (http://www.genome.jp/kegg/brite.html) to obtain their hierarchical classification on various aspects of biological system. The occurrence of KO terms represented in multiple functional levels was recorded.

### Noncoding RNA annotation

Full-length cDNAs that did not encode known proteins were searched for structural similarity against Rfam 10.1 [33] using ‘cmsearch’ tool (E<10^−5^) in Infernal 1.0 [34]. Since “cmsearch” looks for sequences homologous to covariance models, this stringent approach may miss some highly divergent noncoding RNAs. Thus, full-length cDNAs that did not have a match to known RNA from “cmsearch” were further searched against Rfam 10.1 [33] using BlastR [35] with an E-value cutoff of<10^−6^. These cDNAs were also searched against lncRNA databases such as NONCODE and lncRNAdb. Those found to match RNAs in Rfam 10.1 or lncRNA databases from either approaches were annotated as known noncoding RNA.

### Search for tetrapod and teleost fish homologs of elephant shark proteins

The tetrapod and teleost fish homologs of elephant shark full-length proteins were identified by searching elephant shark protein sequences against ENSEMBL proteins of representative tetrapod and teleost fish species using BLASTP (E<10^−5^). The tetrapods included human, mouse (*Mus musculus*), dog (*Canis familiaris*), opossum (*Monodelphis domestica*), platypus (*Ornithorhynchus anatinus*), lizard (*Anolis carolinensis*), chicken (*Gallus gallus*), zebra finch (*Taeniopygia guttata*) and *Xenopus* (*Xenopus tropicalis*) whereas the fishes comprised stickleback (*Gasterosteus aculeatus*), medaka (*Oryzias latipes*), zebrafish (*Danio rerio*) and fugu (*Takifugu rubripes*).

## Results and Discussion

### Cloning and sequencing

Our main objective was to generate full-length cDNA clones and sequences from the elephant shark. To this end, we used the 5′ oligo-capping method [21] that generates full-length mRNA sequences containing a tag at the 5′ end and a polyA tail at the 3′end. Libraries were generated from six adult tissues of elephant shark. To increase the cloning efficiency of the plasmid vector, we selectively cloned mRNA in the range of ∼0.4 kb to 3.5 kb. For each tissue, a total of 13,000–15,000 clones were randomly picked and their 5′ and 3′-ends were sequenced. These sequences were processed and analyzed as described in Methods section and depicted in Figure 1.

Although the oligo-capping method selectively enriches full-length mRNA clones, such libraries are known to contain some 5′ or 3′-end truncated cDNAs. We first looked for such 5′ or 3′ truncated coding sequences by BLASTX searches and categorized them as “ESTs”. These are typically fragments of protein coding sequences and hence are useful in identifying protein-coding genes. Altogether 38,273 such ESTs were identified from various tissues [GenBank: JK927127-JK965399] and classified as “ESTs”. Although they were excluded from further processing (Figure 1), they were annotated by BLASTX searches (see below). We assembled the 5′-end and 3′-end reads of the remaining clones to determine how many of them show a good overlap and to generate full-length cDNA sequences. By using this strategy, we were able to assemble 5,839 full-length protein coding transcripts (483 from gills, 1,410 from intestine, 740 from kidney, 1,024 from liver, 1,749 from spleen and 433 from testis) and 10,701 full-length non-coding transcripts (2,153 from gills, 2,023 from intestine, 2,377 from kidney, 1,119 from liver, 1,330 from spleen and 1,699 from testis). These are typically short-insert clones (average 0.9 kb) containing coding or noncoding full-length cDNAs.

The 5′ and 3′-end reads that did not assemble are likely to contain inserts longer than 1 kb. These reads were clustered using ‘cd-hit-est’ to generate non-redundant sets of 5′-ESTs and 3′-ESTs. The unique sets of 5′-ESTs and 3′-ESTs were then BLASTX searched against the NCBI nr protein database. Based on their BLASTX hits, they were classified as protein-coding and noncoding 5′-ESTs and 3′-ESTs. Note that some of these noncoding ESTs may contain 5′UTR or 3′UTR of protein-coding transcripts. Of the clones whose 5′-ends and 3′-ends showed matches to the amino-terminal and carboxyl-terminal ends of protein sequences respectively, a total of 939 clones (202 from gills, 2 from intestine, 146 from kidney, 187 from liver, 214 from spleen, 188 from testis) were selected for generating full-length protein-coding cDNA sequences by primer walking.

Altogether, we generated 6,778 full-length protein-coding sequences ranging in size from 339 bp to 3,364 bp from the six tissues (Table 1) [GenBank: JX052268-JX053440 and JX207142-JX212746]. In addition 10,701 full-length noncoding sequences [GenBank: JX053441–JX064141] were generated by aligning the 5′- and 3′-end sequences of noncoding clones (Table 1). These should be considered as putative full-length noncoding cDNA since some of them may be 5′ or 3′ truncated similar to the protein coding sequences reported above. After clustering the full-length transcripts as described in Methods, we obtained a non-redundant set of 1,173 full-length protein-coding cDNA (331 from gills, 212 from intestine, 200 from kidney, 139 from liver, 160 from spleen, 131 from testis) and 6,229 nr full-length noncoding cDNA sequences (1,245 from gills, 1,079 from intestine, 1,402 from kidney, 478 from liver, 896 from spleen, 1,129 from testis).

**Table 1:** Full-length cDNA (FLcDNA) generated from various tissues.

Tissue	Total FLcDNA	Protein-coding sequences	Noncoding sequences	
			known	novel
Gills	2,864	711	124	2,029
Intestine	3,442	1,419	98	1,925
Kidney	3,262	885	142	2,235
Liver	2,341	1,222	96	1,023
Spleen	3,285	1,955	54	1,276
Testis	2,285	586	82	1,617
Total	17,479	6,778	590	10,111

In addition to the full-length cDNA dataset, our procedure also generated 30,375 5′-ESTs [GenBank: JK855435 – JK885809] and 41,317 3′-ESTs [GenBank: JK885810 – JK927126]. Their tissue distribution is shown in Table 2.

**Table 2:** Sets of 5′-ESTs and 3′-ESTs generated from various tissues.

Tissue	5'-ESTs	5'-ESTs with similarity to known proteins	3'-ESTs	3'-ESTs with similarity to known proteins	Other ESTs with similarity to known proteins
Gills	4,795	2,082	6,174	1,436	5,043
Intestine	3,271	2,000	3,497	1,372	5,378
Kidney	5,470	2,361	7,401	1,654	6,375
Liver	4,147	3,209	5,653	3,732	6,773
Spleen	4,908	2,845	6,068	2,096	5,688
Testis	7,784	2,233	12,524	2,766	9,016
Total	30,375	14,730	41,317	13,056	38,273

### Polyadenylation signal usage

The analysis of polyadenylation signals in the full-length cDNA dataset of elephant shark can provide insights into the pattern of polyadenylation signal usage by cartilaginous fishes. In human, rat and mouse, 85%, 82% and 71% of mRNAs respectively use one of the two canonical polyadenylation sites (AAUAAA and AUUAAA) [13], [27], [36] and the rest use one of 14 alternative polyadenylation sites. Among ray-finned fishes, 93% and 81% of salmon and catfish mRNAs respectively use one of the two canonical polyadenylation sites [18], [20]. In the elephant shark, polyadenylation sites could be identified in 91% of 1,173 nr full-length protein-coding transcripts and 81% of them used one of the canonical polyadenylation sites, similar to human, rat and catfish mRNAs. The remaining 19% used one of 13 alternative signals (Table 3). Among the 6,229 full-length nr noncoding transcripts, 90% contained recognizable polyadenylation sites with ∼80% corresponding to one of the two canonical polyadenylation sites (Table 3). The most frequently used alternative signals in both protein-coding and noncoding transcripts were UAUAAA and AGUAAA, which are also the most frequently used alternative signals in rat [27]. This shows that the polyadenylation usage in elephant shark is very similar to that in mammals. Furthermore, both protein-coding transcripts and noncoding transcripts in elephant shark use the same proportion of canonical polyadenylation sites. It has been shown that the strength of the polyadenylation signal is correlated to the elongation of the poly(A) tail, which in turn is correlated to translation efficiency [37]. Thus in the elephant shark, both protein-coding transcripts and noncoding transcripts are polyadenylated and translated with the same efficiency, which implies that noncoding transcripts are as important as protein-coding transcripts.

**Table 3:** Types of polyadenylation signals observed in protein-coding and noncoding full-length cDNA transcripts of elephant shark.

Polyadenylation signal type	Protein-coding transcripts		Noncoding transcripts	
	Number	Percentage	Number	Percentage
AAUAAA	754	64.3%	3,825	61.4%
AUUAAA	199	17.0%	1,145	18.4%
AGUAAA	14	1.2%	120	1.9%
UAUAAA	33	2.8%	164	2.6%
UUUAAA	12	1.0%	63	1.0%
CAUAAA	11	0.9%	47	0.8%
AAGAAA	3	0.3%	36	0.6%
AAUACA	9	0.8%	54	0.9%
GAUAAA	10	0.9%	45	0.7%
AAUAUA	9	0.8%	56	0.9%
AAAACA	3	0.3%	16	0.3%
ACUAAA	1	0.1%	19	0.3%
AAUGAA	1	0.1%	22	0.4%
AAAAAG	2	0.2%	13	0.2%
AAUAGA	2	0.2%	7	0.1%
Not identifiable	110	9.4%	597	9.6%

### Functional annotation of full-length protein-coding cDNA

To assign descriptions to the elephant shark protein-coding cDNA, they were searched against the NCBI nr protein database using BLASTX. The top hit generally represents the homolog of the elephant shark protein and indicates the type of the protein. Of the 6,778 full-length cDNAs searched, 21% showed high similarity (the top BLASTX hits) to known cartilaginous fish genes, while 52% and 23% showed high similarity to tetrapod and ray-finned fish (actinopterygians) sequences, respectively. Thus, a majority of these full-length protein-coding sequences (79%) are being identified for the first time in a cartilaginous fish.

**Figure 2:**
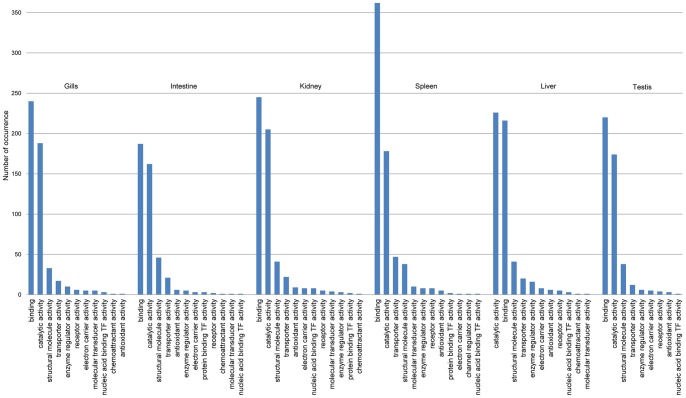
Tissue-wise occurrence of gene ontology terms for full-length protein-coding cDNA (Molecular function).

**Figure 3:**
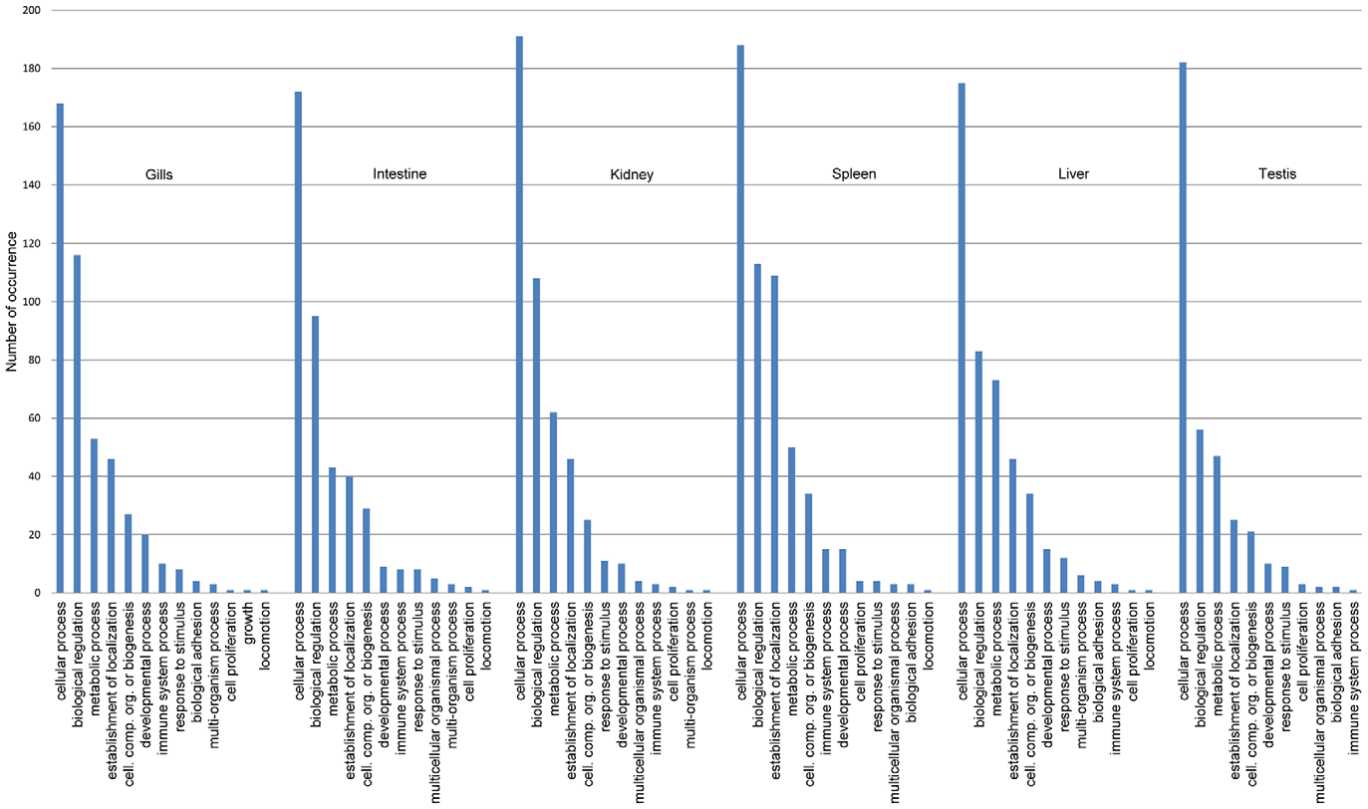
Tissue-wise occurrence of gene ontology terms for full-length protein-coding cDNA (Biological Process).

Further functional annotation of protein sequences was carried out by GO annotation using QuickGO [28]. GO annotation was confined to second levels of only “Molecular Functions” and “Biological Process”. Only 4,985 of the elephant shark proteins were mapped to a UniProt identifier, and of these at least one GO term could be assigned to 4,429 proteins. The number and types of GO terms under “Molecular Function” and “Biological Process” categories across various tissues are shown in Figure 2 and Figure 3, respectively. In the molecular function category, proteins involved in “binding” (GO:0005488), “catalytic activity” (GO:0003824), “structural molecule activity” (GO:0005198) and “transporter activity” (GO:0005215) are the most abundant across all six tissues (Figure 2). In the biological process category, proteins involved in “cellular process” (GO:0009987), “biological regulation” (GO:0065007), “metabolic process” (GO:0008152), and “establishment of localization” (GO:0051234) are among the top four abundant proteins across all tissues (Figure 3). Although “immune system process” (GO:0002376) proteins are expressed in all tissues, their numbers are substantially higher in spleen, intestine and gills, consistent with the immunological roles of these lymphatic tissues in cartilaginous fishes. As alluded above, spleen is the major lymphatic organ in cartilaginous fishes. Moreover, although intestine and gills do not produce immune cells, these organs are known to accumulate immune cells in cartilaginous fishes [38], [39].

We then annotated the protein domains in elephant shark proteins using InterProScan. The top 10 InterPro domains identified in various tissues are given in Table S1. Globin and haemoglobin-related protein domains are the top-most domains in spleen, gills and kidney. This is likely to be due to the high levels of erythrocytes in the gills and kidney owing to their primary role in respiration and osmoregulation, respectively. The venous sinuses of the spleen of cartilaginous fishes are also known to be filled mainly with erythrocytes, unlike those in mammals which are filled primarily with lymph [40]. As expected, the cytochrome P450 superfamily is the most abundant domain in the liver which is the main tissue for the metabolism of toxic compounds catalyzed by cytochrome P450 family of enzymes.

KEGG PATHWAY is a set of manually drawn pathways of molecular interactions that helps in interpreting the biological functions at the systems level. We performed KEGG pathway analysis by KEGG orthology (KO) annotation using the Automatic Annotation Server (KAAS) [32]. Of the 6,778 elephant shark proteins, 4,384 were mapped to a KEGG pathway and their KO terms were categorized into different functional biological systems. The numbers of KO terms across various tissues are shown in Table 4. The KO terms are broadly classified under five functional categories: cellular processes, environmental information processing, genetic information processing, metabolism and organismal systems. Under cellular processes (Table 4A), transport and catabolism are the most represented pathways across the six tissues of the elephant shark while the most common pathway under environmental information processing is signal transduction (Table 4B). Translation is the most common pathway under genetic information processing (Table 4C). Under the metabolism category, proteins involved in energy metabolism pathway are more common in intestine, kidney and liver while those involved in lipid metabolism and xenobiotics biodegradation and metabolism are the most abundant in the liver (Table 4D). At the organismal systems level (Table 4E), proteins involved in circulatory system are common in the intestine and kidney while those involved in the digestive system are most abundant in the intestine. Intestine also has the highest number of endocrine system proteins due to the high incidence of the KO term KO8751 described as the “fatty acid-binding protein 2, intestinal” (data not shown). The liver, gills, spleen and intestine express high numbers of proteins associated with the immune system (Table 4E).

**Table 4:** KEGG ontology categorization for full-length protein-coding cDNA from various tissues.

KEGG categories	Number of KO terms					
	Gills	Intestine	Kidney	Liver	Spleen	Testis
**A. Cellular Processes**	**69**	**40**	**56**	**38**	**61**	**62**
Cell Communication	18	9	11	6	9	19
Cell Growth and Death	6	6	4	2	7	5
Cell Motility	15	9	4	3	5	7
Transport and Catabolism	30	16	37	27	40	31
**B. Environmental Information Processing**	**49**	**43**	**24**	**28**	**55**	**26**
Signal Transduction	29	19	17	23	37	22
Signaling Molecules and Interaction	20	24	7	5	18	2
Membrane Transport	-	-	-	-	-	2
**C. Genetic Information Processing**	**164**	**535**	**228**	**258**	**381**	**257**
Folding, Sorting and Degradation	27	31	21	15	30	28
Replication and Repair	2	1	4	2	1	7
Transcription	6	8	7	8	15	21
Translation	129	495	196	233	335	201
**D. Metabolism**	**98**	**179**	**229**	**267**	**106**	**102**
Amino Acid Metabolism	13	14	24	37	12	11
Biosynthesis of Other Secondary Metabolites	-	2	2	3	3	-
Carbohydrate Metabolism	11	7	29	23	16	21
Energy Metabolism	31	87	88	51	26	22
Glycan Biosynthesis and Metabolism	5	5	6	3	4	2
Lipid Metabolism	10	3	10	51	10	13
Metabolism of Cofactors and Vitamins	6	9	9	15	22	5
Metabolism of Other Amino Acids	10	24	28	30	5	9
Metabolism of Terpenoids and Polyketides	-	1	1	6	-	3
Nucleotide Metabolism	1	4	6	5	2	7
Xenobiotics Biodegradation and Metabolism	11	23	26	43	6	9
**E. Organismal Systems**	**98**	**385**	**107**	**147**	**116**	**68**
Circulatory System	22	53	40	17	5	3
Development	2	-	-	2	4	2
Digestive System	3	162	5	32	26	7
Endocrine System	14	124	26	35	20	14
Environmental Adaptation	2	-	1	1	-	-
Excretory System	5	10	11	5	11	10
Immune System	38	29	15	45	32	16
Nervous System	9	7	8	9	17	12
Sensory System	3	-	1	1	1	4

### Full-length noncoding cDNA sequences

A total of 10,701 full-length noncoding cDNA sequences were generated from the six elephant shark tissues. Of these, 590 sequences are similar to known ncRNA genes in Rfam 10.1 [33]. The types and counts of these ncRNA are shown in Table 5. The majority of these (88%) are housekeeping ncRNAs such as tRNA and rRNA. Only one of them is a miRNA, mir-598, which is expressed in all the elephant shark tissues analyzed. This miRNA has been previously identified only in tetrapods (see [41]–[42]) but not in teleost fishes or in jawless vertebrates [43]. Its presence in elephant shark indicates that this is an ancestral vertebrate miRNA that has been either lost or yet to be identified in teleost fishes. In mammals, a large number of long ncRNAs have been shown to be transcribed in the anti-sense strand of protein-coding genes [44]. Of the 10,111 noncoding elephant shark cDNA, 260 showed similarity (E<10−7) to partial protein sequences on the anti-sense strand, indicating that antisense transcripts are also common in cartilaginous fishes.

**Table 5:** Families of ncRNA genes present in various tissues of elephant shark.

RNA type	Number
5_8S_rRNA	2
SSU_rRNA_eukarya	135
tRNA	386
Mir-598	15
snoRNA	11
U1	3
U2	3
U3	2
U4	9
U5	2
U6	1
7SK	11
Clostridiales-1 RNA	6
CsrB/RsmB RNA family	1
Metazoa_SRP	3

A vast majority of elephant shark noncoding cDNA has no similarity to known ncRNAs. These are unlikely to be artefacts such as genomic DNA remnants, because we had treated elephant shark RNA with DNAse at two stages in the preparation of cDNA. These noncoding cDNAs are either highly divergent ncRNA or novel ncRNA with no known orthologs in other vertebrates. Unlike protein coding sequences that generally show high level of sequence conservation across even evolutionarily distant species, ncRNA are known to exhibit only modest sequence conservation [45]. Thus, it is possible that many of these ‘unknown’ elephant shark ncRNA have orthologs in other species that are quite divergent at the sequence level.

### Elephant shark genes differentially lost in tetrapods and teleost fishes

Since cartilaginous fishes are the most basal phylogenetic group of jawed vertebrates, genes shared between elephant shark and bony vertebrates represent ancient genes that were present in the common ancestor of jawed vertebrates. Consequently, elephant shark genes can be used to determine if any of the ancestral genes have been differentially lost during the evolution of tetrapod and teleost lineages. To identify such differentially lost genes, we searched the elephant shark full-length protein sequences against the ENSEMBL proteins of tetrapods (human, mouse, dog, opossum, platypus, lizard, chicken, zebra finch and *Xenopus*) and teleost fishes (zebrafish, stickleback, medaka and fugu) using BLASTP (E<10−5). Proteins that had hits in one group but not in the other group were further searched against NCBI nr protein database to confirm that they do not have any orthologs in the respective groups. This analysis identified six elephant shark genes that have orthologs in tetrapods but not in teleost fishes. The expression patterns and known function of the mouse orthologs of these elephant shark genes are given in Table 6. Three of these genes, *Igj*, *Mt4* and *Tigd4* belong to multigene families and therefore it is possible that they were redundant in teleost fishes. However, the other three genes, *A4gnt*, *Hmgn1* and *Clps* appear to be essential genes in mouse as revealed by the phenotypes of knockout or mutant mice (see Table 6). The loss of these genes in teleosts might have been compensated by recruitment of new genes to perform their functions or resulted in altered phenotypes in teleost fishes.

**Table 6:** Ancient genes differentially lost in teleost fishes.

Elephant shark GenBank accession	Expression pattern of elephant shark gene	Mouse ID/ Gene	Description	Function in mouse	GO term (Biological Process)	Protein domains	Expression data
JX052286	Gills	MGI:2143261 *A4gnt*	Alpha-1,4-N-acetylglucosaminyl-transferase	Mice homozygous for a knock-out allele exhibit gastric adenocarcinoma with increased cell proliferation, angiogenesis, inflammation and gastric mucosal thickness.	Glycoprotein biosynthetic process	Alpha 1,4-glycosyltransferase domain; Glycosyltransferase, DXD sugar-binding motif	Gastric gland mucous cells, duodenal Brunner's glands
JX052312	Gills, intestine, spleen	MGI:96493 *Igj*	Immunoglobulin joining chain	Formation of polymeric Igs and their transport into secretions	Humoral immune response	Immunoglobulin J chain	Blood, intestine, spleen
JX052629	All six tissues	MGI:96120 *Hmgn1*	High mobility group nucleosomal binding domain 1	Mice homozygous for a knock-out allele display partial embryonic lethality, increased cellular sensitivity to ultraviolet- and gamma-irradiation, increased tumor incidence and metastatic potential, increased incidence of ionizing radiation-induced tumors, and abnormal cell cycle checkpoint function.	Chromatin organization, post-embryonic camera-type eye morphogenesis, etc.	High mobility group nucleosome-binding domain-containing family	Ubiquitous expression
JX052670	Intestine, kidney, liver	MGI:99692 *Mt4*	Metallothionein 4	Organization and assembly of metal- thiolate clusters	Ccellular metal ion homeostasis, response to cadmium ion	Metallothionein domain, vertebrate; Metallothionein superfamily, eukaryotic	Decidua, embryo, liver, tongue
JX052805	Intestine	MGI:88421 *Clps*	Colipase, pancreatic	Homozygous mutation of this gene results in increased mortality before weaning. Surviving mutants are growth retarded and remain smaller than wild-type into adulthood with decreased body fat, impaired fat absorption, elevated cholesterol, and reduced triglycerides.	Digestion, lipid catabolic process, etc.	Colipase; Colipase, conserved site; Colipase, C-terminal; Colipase, N-terminal	Pancreas
JX053121	Liver	MGI:2685264 *Tigd4*	Tigger transposable element derived 4	Regulation of transcription	Biological process	DDE superfamily endonuclease, CENP-B-like; DNA binding HTH domain, Psq-type; Homeodomain-like; HTH CenpB-type DNA-binding domain	Diaphragm, tongue, skeletal muscle, vertebral axis muscle system

Four of the analyzed elephant shark full-length proteins had orthologs in teleost fishes but not in tetrapods. The descriptions, protein domains and GO terms associated with zebrafish orthologs of the four elephant shark genes are given in Table 7. Two of the elephant shark genes (GenBank JX052420 and JX053182) have only a single ortholog in zebrafish, *CCL-C24j*. The proteins encoded by the two elephant shark genes show only 41% identity to each other and are likely to be the result of a lineage-specific gene duplication in the elephant shark. These elephant shark and zebrafish chemokine genes could be involved in immune response unique to the aquatic habitat of elephant shark and teleost fishes. Of the two other elephant shark genes, one codes for a hypothetical gene related to the caspase family (JX052809) and the other codes for a FYVE and coiled-coil domain (JX052773). The function and expression patterns of the zebrafish orthologs of these genes are currently unknown.

**Table 7:** Ancient genes differentially lost in tetrapods.

Elephant shark GenBank accession	Expression pattern of elephant shark gene	Zebrafish ID/ Gene	Description	Function in zebrafish	GO term (Biological Process)	Protein domains	Expression data
JX052809	Intestine	ZDB-GENE-081104-302 si:dkey-10c21.1	Hypothetical protein	Not known	Regulation of apoptotic process	CARD; Caspase activation and recruitment domain: a protein-protein interaction domain DD_superfamily; The Death Domain Superfamily of protein-protein interaction domains	Not known
JX052773	Intestine	ZDB-GENE-050419-230 si:dkey-246j7.1	FYVE and coiled-coil domain-containing protein 1	Not known	Not known	FYVE domain; Zinc-binding domain; targets proteins to membrane lipids via interaction with phosphatidylinositol-3-phosphate, PI3P; present in Fab1, YOTB, Vac1, and EEA1; SMC_prok_B; chromosome segregation protein SMC, common bacterial type; RUN domain	Not known
JX052420	Gills, intestine, spleen	ZDB-GENE-091204-344 si:dkey-25o1.5	Chemokine CCL-C24j	Small cytokines, including a number of secreted growth factors and interferons involved in mitogenic, chemotactic, and inflammatory activity; distinguished from other cytokines by their receptors, which are G-protein coupled receptors; divided.	Immune response	Chemokine interleukin-8-like domain	Not known
JX053182	Spleen						

Our analysis also identified a unique elephant shark gene [GenBank: JX052984] that codes for a 300 amino acid protein with a S-adenosylmethionine-dependent methyltransferase (AdoMet-MTase) domain. This gene has no ortholog in bony vertebrates but shows high similarity (BLASTP E-values 10^−105^ to 10^−92^) to hypothetical proteins predicted in several invertebrates such as the amphioxus (*Branchiostoma floridae*), sea squirt (*Ciona intestinalis*), sea urchin *(Strongylocentrotus purpuratus),* acorn worm (*Saccoglossus kowalevskii*), sea anemone (*Nematostella vectensis*) and the placozoan, *Trichoplax adhaerens*. This is clearly an ancient metazoan gene that has been retained in invertebrates and elephant shark but lost in bony vertebrates. It would be interesting to investigate the function of this gene which seems to be important for the biology of invertebrates and cartilaginous fishes but not for bony vertebrates.

### Functional annotation of 5′-ESTs and 3′-ESTs

The large number of 5′ESTs and 3′ESTs sequenced from various tissues of elephant shark represents a random set of transcripts and hence provides an indication of the expression profile of genes across the tissues. We analyzed these ESTs by first searching them against the NCBI nr protein database using BLASTX. Out of the 30,375 5′-ESTs and 41,317 3′-ESTs, 14,730 5′-ESTs (48.5%) and 13,056 3′-ESTs (31.6%) code for known proteins. The remaining 5′-ESTs (15,645) and 3′-ESTs (28,261) represent noncoding transcripts or UTRs of protein-coding transcripts. For functional annotation of coding 5′- and 3′-ESTs, we considered only the 5′-EST sequence if our set included both 5′ and 3′-EST of a clone. In addition, all singleton protein-coding 5′- and 3′-ESTs were included. This set added up to a combined set of 21,964 unique 5′ and 3′ ESTs (or cDNA clones), from six elephant shark tissues. Of these 21,964 5′ and 3′-ESTs, 7% showed high similarity (the top BLASTX hits) to cartilaginous fish genes, while 64% and 26% showed high similarity to tetrapod and ray-finned fish genes, respectively.

The combined set of 5′ and 3′-ESTs was functionally annotated by analyzing GO terms associated with them using GOstat [30]. GO terms obtained were further analyzed for significant enrichment (p-value <0.05) in each tissue. Of the 21,964 combined set of 5′-ESTs and 3′-ESTs, 12,582 mapped to a UniProt identifier. 11,154 of these ESTs were assigned to one or more GO terms. The top ten enriched GO terms under “Molecular Function” and “Biological Process” categories across various tissues are shown in Table S2 and S2, respectively. In the molecular function category, proteins involved in binding (e.g., GTP binding, ion binding, actin binding) and catalytic activity are significantly enriched across all six tissues (Table S2). In the biological process category, there was an enrichment of metabolic process and cellular process across all six tissues (Table S3). In addition, under the molecular function category, proteins with chemokine activity are significantly enriched in gills, intestine, and spleen (Table S2) which is consistent with the accumulation or production of immune cells in these tissues of cartilaginous fishes [38], [39]. Intestine and spleen were also found to be enriched in immune response proteins (biological process) (Table S3). Proteins associated with blood coagulation, platelet activation and complement activation were uniquely enriched in the liver (Table S3). Proteins associated with mitotic cell division are enriched in the testis (Table S3), spleen (p-value <0.002) and intestine (p-value <0.002) whereas those involved in meiosis are uniquely enriched in testis (p-value <0.005) (not shown in Table S3).

The proteins encoded by the elephant shark 5′-EST and 3′-ESTs were further analysed for InterPro domains. The top ten InterPro protein domains identified are shown in Table S4. The domain for “Protein synthesis factor, GTP-binding” is one of the most abundant domains across all the six tissues. The various domains of the translation elongation factor EFTu/EF1A are also among the abundant domains in all tissues except the liver. In liver, the highest count was for the various domains of vitellogenin, a precursor of egg-yolk protein which is known to be highly expressed in the liver of females during the spawning season.

### Functional annotation of other ESTs

Although we used the “oligo-capping” method to selectively enrich full-length cDNA clones in the library, we encountered a large number (38,273) of 5′ or 3′-end truncated protein-coding ESTs (see “Cloning and sequencing” section above). These are all protein-coding ESTs and hence useful for identifying genes in the elephant shark genome. We annotated these ESTs by BLASTX search against NCBI nr protein database. Of the 38,273 ESTs, 3% (1,226) showed high similarity (the top BLASTX hits) to cartilaginous fish genes, 71% (25,221) and 21% (7,539) showed high similarity to tetrapod and ray-finned fish (actinopterygians) sequences, respectively. Altogether, these ESTs correspond to 12,192 unique protein identifiers in the NCBI nr database. Thus, these ESTs allow interrogation of a large number of genes (e.g, probing a BAC library, expression profiling) in the elephant shark genome.

### Conclusion

The elephant shark has the smallest known genome (∼910 Mb) among cartilaginous fishes and hence it is a useful model cartilaginous fish genome [5], [6]. Its whole genome is currently being sequenced with funding from the National Institutes of Health, USA (http://esharkgenome.imcb.a-star.edu.sg/). In this study, we have generated and sequenced cDNA libraries enriched in full-length cDNA sequences from six tissues of the elephant shark. In total we sequenced 6,778 full-length protein-coding cDNA and 10,701 full-length noncoding cDNA from six tissues. These represent a unique set of 1,173 full-length coding cDNA sequences and 6,229 full-length noncoding sequences of the elephant shark. BlastX searches of the coding cDNA sequences showed that 79% of them are being identified for the first time in a cartilaginous fish. These full-length coding as well as noncoding sequences are useful resources for annotating coding and noncoding genes in the genomes of elephant shark as well as other cartilaginous fishes. In addition, the clones of full-length coding sequences are valuable resources for functional studies of proteins encoded by them.

In addition to full-length cDNA sequences, we also sequenced a large number of 5′-ESTs (30,375) and 3-‘ESTs (41,317) from the six tissues of the elephant shark. Approximately 50% of these ESTs (21,964 cDNA clones) code for proteins. A major challenge in annotating whole genome sequences is the accurate prediction of transcriptional start sites, 5′UTRs and 3′-UTRs in the whole genome sequences. Currently there are no efficient *in silico* methods that can accurately predict these important features of genes in cartilaginous fishes for which very little genomic resources are available. Most of the prediction methods developed are meant for well characterized genomes of mammals such as human and mouse [46], [47]. The 5′-ESTs and 3′-ESTs generated in our study using the ‘oligo-capping’ method would thus be invaluable tools for precisely predicting the transcription start sites, 5′UTRs and 3′UTRs in the whole genome sequence of the elephant shark. The clones of these ESTs will be useful for obtaining full-length sequences of genes, if required. Thus, besides identifying a large number of coding and noncoding genes in the elephant shark genome, our study has generated genomic resources that would be useful for annotating coding and noncoding sequences in the genome of elephant shark as well as other cartilaginous fishes.

## Supporting Information

Table S1 Top ten InterPro domains identified in the full-length cDNA from various tissues.
(RTF)

Table S2 GO terms (Molecular Function) enriched in the 5′-ESTs and 3′-ESTs from various tissues of elephant shark.
(RTF)

Table S3 GO terms (Biological Process) enriched in the 5′-ESTs and 3′-ESTs from various tissues of elephant shark.
(RTF)

Table S4 Top ten InterPro domains identified in 5′-ESTs and 3′-ESTs from various tissues of elephan shark.
(RTF)
