# Supplementary material for: Sequencing and Analysis of Full-Length cDNAs, 5′-ESTs and 3′-ESTs from a Cartilaginous Fish, the Elephant Shark (Callorhinchus milii)
Source: PLoS One. 2012 Oct 8;7(10):e47174. doi: 10.1371/journal.pone.0047174 (PMC3466250; doi:10.1371/journal.pone.0047174)
Supplement: Table S1 — Top ten InterPro domains identified in the full-length cDNA from various tissues. (RTF) [file pone.0047174.s001.rtf]

Table S1. Top ten InterPro domains identified in the full-length cDNA from various tissues.

Tissues	Protein domains	Number of occurrences	
Gills	Globin, structural domain	16	
	Haemoglobin, alpha	12	
	Cytochrome P450	10	
	Chemokine interleukin-8-like domain	10	
	Proteasome, subunit alpha/beta	10	
	Short-chain dehydrogenase/reductase SDR	9	
	Peptidase M17	8	
	ATPase, F1/A1 complex, alpha/beta subunit, N-terminal	8	
	Small GTPase superfamily	8	
	Tubulin/FtsZ, 2-layer sandwich domain	8	
Intestine	Cytosolic fatty-acid binding	21	
	Proteasome, subunit alpha/beta	20	
	Nucleoside diphosphate kinase	18	
	Proteasome, alpha-subunit, N-terminal domain	10	
	Proteasome, beta-type subunit, conserved site	10	
	Ribosomal protein L5 eukaryotic/L18 archaeal	9	
	NAD(P)-binding domain	9	
	Alkyl hydroperoxide reductase subunit C/ Thiol specific antioxidant	9	
	Small GTPase superfamily	8	
	Globin, structural domain	8	
Kidney	Globin, structural domain	28	
	Ferritin/ribonucleotide reductase-like	18	
	Ferritin	18	
	Lactate dehydrogenase/glycoside hydrolase, family 4, C-terminal	16	
	Globin, subset	14	
	Thioredoxin	12	
	Homeodomain	12	
	L-lactate/malate dehydrogenase	12	
	Haemoglobin, alpha	12	
	Ferritin/DPS protein domain	12	
Spleen	Globin, structural domain	148	
	Haemoglobin, alpha	100	
	Globin, subset	74	
	Haemoglobin, beta	48	
	Proteasome, subunit alpha/beta	18	
	Ferritin/ribonucleotide reductase-like	12	
	Proteasome, alpha-subunit, N-terminal domain	12	
	Ferritin	12	
	Short-chain dehydrogenase/reductase SDR	11	
	Small GTPase superfamily	10	
Liver	Cytochrome P450	20	
	Proteasome, subunit alpha/beta	10	
	AAA+ ATPase domain	10	
	Ferritin/ribonucleotide reductase-like	9	
	Ribosomal protein L5 eukaryotic/L18 archaeal	9	
	Ferritin	9	
	ATPase, F1/A1 complex, alpha/beta subunit, N-terminal	8	
	Cytochrome P450, E-class, CYP3A	8	
	Cytochrome P450, E-class, group I	8	
	Cytochrome P450, E-class, group II	8	
Testis	Tubulin/FtsZ, 2-layer sandwich domain	12	
	Lactate dehydrogenase/glycoside hydrolase, family 4, C-terminal	12	
	Tubulin/FtsZ, C-terminal	12	
	Short-chain dehydrogenase/reductase SDR	11	
	NAD(P)-binding domain	10	
	Protein synthesis factor, GTP-binding	9	
	L-lactate/malate dehydrogenase	9	
	Cyclophilin-like peptidyl-prolyl cis-trans isomerase domain	9	
	Cyclophilin-type peptidyl-prolyl cis-trans isomerase, conserved site	9	
	Small GTPase superfamily	8	
