# Supplementary material for: Sequencing and Analysis of Full-Length cDNAs, 5′-ESTs and 3′-ESTs from a Cartilaginous Fish, the Elephant Shark (Callorhinchus milii)
Source: PLoS One. 2012 Oct 8;7(10):e47174. doi: 10.1371/journal.pone.0047174 (PMC3466250; doi:10.1371/journal.pone.0047174)
Supplement: Table S2 — GO terms (Molecular Function) enriched in the 5′-ESTs and 3′-ESTs from various tissues of elephant shark. (RTF) [file pone.0047174.s002.rtf]

Table S2. GO terms (Molecular Function) enriched in the 5'-ESTs and 3'-ESTs from various tissues. 

	GO term	Description	p-value	
Gills	GO:0003824	catalytic activity	0.00E+00	
	GO:0005525	GTP binding	1.96E-12	
	GO:0005509	calcium ion binding	2.28E-07	
	GO:0005544	calcium-dependent phospholipid binding	3.49E-06	
	GO:0051082	unfolded protein binding	4.37E-06	
	GO:0003924	GTPase activity	8.68E-06	
	GO:0004867	serine-type endopeptidase inhibitor activity	1.21E-04	
	GO:0008009	chemokine activity	1.84E-04	
	GO:0004859	phospholipase inhibitor activity	3.32E-04	
	GO:0003779	actin binding	3.62E-04	
Intestine	GO:0004111	creatine kinase activity	2.81E-09	
	GO:0003779	actin binding	9.22E-07	
	GO:0008097	5S rRNA binding	2.69E-06	
	GO:0005525	GTP binding	2.99E-06	
	GO:0016712	oxidoreductase activity	3.24E-06	
	GO:0008289	lipid binding	1.03E-05	
	GO:0003735	structural constituent of ribosome	2.85E-05	
	GO:0008430	selenium binding	9.31E-05	
	GO:0008009	chemokine activity	1.49E-04	
	GO:0005047	signal recognition particle binding	3.36E-04	
Kidney	GO:0005488	binding	0.00E+00	
	GO:0003824	catalytic activity	0.00E+00	
	GO:0005525	GTP binding	5.31E-05	
	GO:0005332	gamma-aminobutyric acid:sodium symporter activity	8.18E-05	
	GO:0008430	selenium binding	1.98E-04	
	GO:0003924	GTPase activity	2.54E-04	
	GO:0005516	calmodulin binding	7.48E-04	
	GO:0008191	metalloendopeptidase inhibitor activity	7.48E-04	
	GO:0003884	D-amino-acid oxidase activity	1.25E-03	
	GO:0033981	D-dopachrome decarboxylase activity	1.38E-03	
Liver	GO:0005319	lipid transporter activity	2.33E-15	
	GO:0008199	ferric iron binding	1.12E-13	
	GO:0016712	oxidoreductase activity	3.83E-13	
	GO:0004867	serine-type endopeptidase inhibitor activity	3.81E-12	
	GO:0003735	structural constituent of ribosome	6.02E-11	
	GO:0005509	calcium ion binding	4.13E-09	
	GO:0047150	betaine-homocysteine S-methyltransferase activity	5.61E-08	
	GO:0030674	protein binding, bridging	7.86E-08	
	GO:0004252	serine-type endopeptidase activity	1.18E-07	
	GO:0004497	monooxygenase activity	5.81E-06	
Spleen	GO:0005488	binding	0.00E+00	
	GO:0003824	catalytic activity	0.00E+00	
	GO:0005344	oxygen transporter activity	3.70E-14	
	GO:0019825	oxygen binding	2.00E-12	
	GO:0005525	GTP binding	3.38E-12	
	GO:0003735	structural constituent of ribosome	9.89E-08	
	GO:0003924	GTPase activity	1.71E-07	
	GO:0005094	Rho GDP-dissociation inhibitor activity	6.37E-06	
	GO:0004298	threonine-type endopeptidase activity	1.85E-05	
	GO:0016494	C-X-C chemokine receptor activity	6.56E-05	
Testis	GO:0005488	binding	0.00E+00	
	GO:0003824	catalytic activity	0.00E+00	
	GO:0005525	GTP binding	9.64E-12	
	GO:0051082	unfolded protein binding	3.35E-09	
	GO:0003924	GTPase activity	3.81E-09	
	GO:0003743	translation initiation factor activity	2.15E-06	
	GO:0017127	cholesterol transporter activity	3.99E-06	
	GO:0005047	signal recognition particle binding	3.99E-06	
	GO:0005094	Rho GDP-dissociation inhibitor activity	1.01E-05	
	GO:0004809	tRNA (guanine-N2-)-methyltransferase activity	3.14E-05	
