# Supplementary material for: Sequencing and Analysis of Full-Length cDNAs, 5′-ESTs and 3′-ESTs from a Cartilaginous Fish, the Elephant Shark (Callorhinchus milii)
Source: PLoS One. 2012 Oct 8;7(10):e47174. doi: 10.1371/journal.pone.0047174 (PMC3466250; doi:10.1371/journal.pone.0047174)
Supplement: Table S3 — GO terms (Biological Process) enriched in the 5′-ESTs and 3′-ESTs from various tissues of elephant shark. (RTF) [file pone.0047174.s003.rtf]

Table S3. GO terms (Biological Process) enriched in the 5'-ESTs and 3'-ESTs from various tissues.

	GO term	Description	p-value	
Gills	GO:0008152	metabolic process	0.00E+00	
	GO:0007018	microtubule-based movement	7.56E-07	
	GO:0038032	termination of G-protein coupled receptor signaling pathway	9.66E-07	
	GO:0051258	protein polymerization	1.66E-05	
	GO:0006184	GTP catabolic process	3.99E-05	
	GO:0007017	microtubule-based process	4.79E-05	
	GO:0007264	small GTPase mediated signal transduction	1.19E-04	
	GO:0006839	mitochondrial transport	4.63E-04	
	GO:0006457	protein folding	8.53E-04	
	GO:0045751	negative regulation of Toll signaling pathway	1.12E-03	
Intestine	GO:0008152	metabolic process	0.00E+00	
	GO:0009987	cellular process	0.00E+00	
	GO:0030833	regulation of actin filament polymerization	2.83E-04	
	GO:0006955	immune response	4.91E-04	
	GO:0006635	fatty acid beta-oxidation	5.83E-04	
	GO:0006869	lipid transport	9.32E-04	
	GO:0030043	actin filament fragmentation	1.19E-03	
	GO:0007264	small GTPase mediated signal transduction	1.56E-03	
	GO:0046439	L-cysteine metabolic process	2.06E-03	
	GO:0050713	negative regulation of interleukin-1 beta secretion	2.17E-03	
Kidney	GO:0008152	metabolic process	0.00E+00	
	GO:0044237	cellular metabolic process	0.00E+00	
	GO:0009987	cellular process	0.00E+00	
	GO:0000050	urea cycle	5.28E-06	
	GO:0015991	ATP hydrolysis coupled proton transport	1.38E-05	
	GO:0007018	microtubule-based movement	2.81E-05	
	GO:0000381	regulation of alternative nuclear mRNA splicing, via spliceosome	4.37E-05	
	GO:0006096	glycolysis	1.99E-04	
	GO:0006596	polyamine biosynthetic process	2.17E-04	
	GO:0051258	protein polymerization	4.44E-04	
Liver	GO:0008152	metabolic process	0.00E+00	
	GO:0007596	blood coagulation	3.37E-21	
	GO:0006869	lipid transport	2.84E-12	
	GO:0006879	cellular iron ion homeostasis	4.35E-12	
	GO:0006826	iron ion transport	1.69E-11	
	GO:0030168	platelet activation	7.83E-11	
	GO:0010951	negative regulation of endopeptidase activity	1.71E-06	
	GO:0000050	urea cycle	1.75E-06	
	GO:0006956	complement activation	4.88E-06	
	GO:0051258	protein polymerization	5.83E-06	
Spleen	GO:0008152	metabolic process	0.00E+00	
	GO:0009987	cellular process	0.00E+00	
	GO:0015671	oxygen transport	5.12E-12	
	GO:0006955	immune response	2.30E-09	
	GO:0006184	GTP catabolic process	1.34E-06	
	GO:0007264	small GTPase mediated signal transduction	2.03E-06	
	GO:0030833	regulation of actin filament polymerization	1.31E-05	
	GO:0043249	erythrocyte maturation	1.85E-05	
	GO:0007018	microtubule-based movement	2.12E-05	
	GO:0019885	antigen processing and presentation of endogenous peptide antigen via MHC class I	1.11E-04	
Testis	GO:0008152	metabolic process	0.00E+00	
	GO:0007067	mitosis	7.99E-16	
	GO:0006184	GTP catabolic process	4.81E-08	
	GO:0007018	microtubule-based movement	9.72E-08	
	GO:0006457	protein folding	2.13E-07	
	GO:0007093	mitotic cell cycle checkpoint	1.12E-06	
	GO:0051258	protein polymerization	2.90E-06	
	GO:0006413	translational initiation	7.89E-06	
	GO:0034501	protein localization to kinetochore	9.72E-06	
	GO:0007017	microtubule-based process	1.02E-05	
