# Supplementary material for: Sequencing and Analysis of Full-Length cDNAs, 5′-ESTs and 3′-ESTs from a Cartilaginous Fish, the Elephant Shark (Callorhinchus milii)
Source: PLoS One. 2012 Oct 8;7(10):e47174. doi: 10.1371/journal.pone.0047174 (PMC3466250; doi:10.1371/journal.pone.0047174)
Supplement: Table S4 — Top ten InterPro domains identified in 5′-ESTs and 3′-ESTs from various tissues of elephan shark. (RTF) [file pone.0047174.s004.rtf]

Table S4. Top ten InterPro domains identified in 5'-ESTs and 3'-ESTs from various tissues.

Tissue	Protein domain	Number of occurrences	
Gills	Protein synthesis factor, GTP-binding	294	
	Translation elongation factor EF1A, eukaryotic/archaeal	258	
	Tubulin/FtsZ, C-terminal	100	
	Translation elongation factor EFTu/EF1A, domain 2	98	
	Tubulin/FtsZ, 2-layer sandwich domain	96	
	Peptidase C74, NS2 pestivirus	96	
	Translation elongation factor EFTu/EF1A, C-terminal	88	
	Alpha tubulin	84	
	Helicase, C-terminal	72	
	Protein-tyrosine phosphatase, active site	72	
Intestine	Protein synthesis factor, GTP-binding	294	
	Translation elongation factor EF1A, eukaryotic/archaeal	267	
	Cytochrome P450	145	
	Cytochrome P450, E-class, group II	116	
	Cytochrome P450, E-class, CYP3A	116	
	Translation elongation factor EFTu/EF1A, domain 2	98	
	ATP:guanido phosphotransferase active site	90	
	ATP:guanido phosphotransferase, catalytic domain	90	
	ATP:guanido phosphotransferase, N-terminal	90	
	Translation elongation factor EFTu/EF1A, C-terminal	89	
Kidney	Protein synthesis factor, GTP-binding	255	
	Translation elongation factor EF1A, eukaryotic/archaeal	213	
	Alpha tubulin	174	
	ATPase, F1/A1 complex, alpha/beta subunit, N-terminal	164	
	ATPase, F1/V1/A1 complex, alpha/beta subunit, N-terminal	164	
	Tubulin/FtsZ, 2-layer sandwich domain	128	
	Tubulin/FtsZ, C-terminal	128	
	Tubulin	120	
	ATPase, F1 complex, alpha subunit	111	
	Translation elongation factor EFTu/EF1A, domain 2	85	
Liver	Fibrinogen, alpha/beta/gamma chain, coiled coil domain	670	
	Protein synthesis factor, GTP-binding	327	
	Vitellinogen, beta-sheet shell	302	
	Lipid transport protein, beta-sheet shell	302	
	Vitellinogen, beta-sheet N-terminal	300	
	Vitellinogen, superhelical	300	
	Vitellinogen, open beta-sheet, subdomain 1	300	
	Vitellinogen, open beta-sheet	300	
	Lipid transport protein, N-terminal	300	
	Vitellinogen, open beta-sheet, subdomain 2	300	
Spleen	Protein synthesis factor, GTP-binding	333	
	Translation elongation factor EF1A, eukaryotic/archaeal	273	
	Globin, structural domain	212	
	Aminoacyl-tRNA synthetase, class II	168	
	Aminoacyl-tRNA synthetase, class II (D/K/N)-like	164	
	Aminoacyl-tRNA synthetase, class II (D/K/N)	164	
	Haemoglobin, beta	136	
	Aspartyl/Asparaginyl-tRNA synthetase, class IIb	136	
	Asparaginyl-tRNA synthetase, class IIb	128	
	Translation elongation factor EFTu/EF1A, domain 2	111	
Testis	Protein synthesis factor, GTP-binding	390	
	Tubulin/FtsZ, 2-layer sandwich domain	312	
	Tubulin/FtsZ, C-terminal	312	
	Translation elongation factor EF1A, eukaryotic/archaeal	270	
	Tubulin	192	
	Chaperonin TCP-1, conserved site	183	
	Alpha tubulin	162	
	Chaperonin Cpn60/TCP-1	140	
	Translation elongation factor EFTu/EF1A, domain 2	131	
	Beta tubulin	126	
